# Attention Diversion Improves Response Inhibition of Immediate Reward, But Only When it Is Beneficial: An fMRI Study

**DOI:** 10.3389/fnhum.2016.00429

**Published:** 2016-08-26

**Authors:** Franco Scalzo, David A. O’Connor, Catherine Orr, Kevin Murphy, Robert Hester

**Affiliations:** ^1^Melbourne School of Psychological Sciences, University of MelbourneMelbourne, VIC, Australia; ^2^Cognitive Neuroscience Centre, Reward and Decision-Making Group, Centre National pour la Recherche ScientifiqueLyon, France; ^3^Departments of Psychiatry and Psychology, University of VermontBurlington, VT, USA; ^4^Cardiff University Brain Research Imaging Centre, School of Psychology, Cardiff UniversityCardiff, UK

**Keywords:** attention, response inhibition, reward, Go/No-go task, inferior frontal gyrus, superior temporal gyrus, fMRI

## Abstract

Deficits of self-control are associated with a number of mental state disorders. The ability to direct attention away from an alluring stimulus appears to aid inhibition of an impulsive response. However, further functional imaging research is required to assess the impact of shifts in attention on self-regulating processes. We varied the level of attentional disengagement in an functional magnetic resonance imaging (fMRI)-based Go/No-go task to probe whether diversion of attention away from alluring stimuli facilitates response inhibition. We used the attention-grabbing characteristic of faces to exogenously direct attention away from stimuli and investigated the relative importance of attention and response inhibition mechanisms under different delayed reward scenarios [i.e., where forgoing an immediate reward ($1) led to a higher ($10) or no payoff in the future]. We found that diverting attention improved response inhibition performance, but only when resistance to an alluring stimulus led to delayed reward. Region of interest analyses indicated significant increased activity in posterior right inferior frontal gyrus during successful No-go trials for delayed reward trials compared to no delayed reward trials, and significant reduction in activity in the superior temporal gyri and left caudate in contexts of high attentional diversion. Our findings imply that strategies that increase the perceived benefits of response inhibition might assist individuals in abstaining from problematic impulsive behaviors.

## Introduction

An important facet of human cognition is an individual’s capacity to exercise self-control. The ability to refrain from inappropriate behaviors facilitates effective interaction with society, care for one’s self, and pursuit of longer-term goals at the expense of immediate gratification (Mischel et al., 1988; Baumeister and Heatherton, 1996). Deficits in self-control are associated with a number of health issues including substance abuse (Fillmore and Rush, 2002; Monterosso et al., 2005; Goudriaan et al., 2006) and mental state disorders (Penadés et al., 2007; Boonstra et al., 2010). Self-control has been characterized as a balance between long-term goals and immediate temptations (Ochsner and Gross, 2005; Li and Sinha, 2008; Heatherton and Wagner, 2011). There is also evidence that visual attention is important for controlling impulsive responses to immediately available rewards (Mischel and Ebbesen, 1970; Mischel et al., 1972; Casey et al., 2011). For instance, resistance to a tempting stimulus can be prolonged by focussing on less tempting features of the target (Mischel and Baker, 1975), or deploying attention away from the target (Mischel and Ebbesen, 1970; Mischel et al., 1972). Conversely, by controlling visual fixations toward appetitive items, attention has been shown to bias choices for fixated items (Armel et al., 2008; Lim et al., 2011). However, despite the wealth of research in this field, the question of whether inhibition of motor responses over immediately available rewards can be improved by diverting attention away from them has not yet been explored. Moreover the neural correlates of such a phenomenon remain unexamined.

Response inhibition has been functionally well-defined in the prefrontal cortex (PFC), particularly the right inferior frontal gyrus (rIFG; Aron, 2011; Tabibnia et al., 2011), although others suggest a more general role for the rIFG (Hampshire and Sharp, 2015). On the other hand, the dorsal striatum is implicated in reward-related motivational and learning processes for goal-directed behavior (Balleine et al., 2007; Padmala and Pessoa, 2010; O’Connor et al., 2012). It is theorized that self-regulatory failure is more likely when a striatal response prevails over a PFC response (Heatherton and Wagner, 2011). For instance, exposure to highly alluring cues (e.g., offered your favorite chocolate) may overwhelm PFC response, or PFC function may be impaired (e.g., due to negative mood; Peters and Büchel, 2011). Is it also possible that, by reducing the saliency of stimuli and related striatal response, the probability of self-regulatory failure can be decreased? Hypoactivity in reward-related neural areas has been accompanied by hypoactivity in neural regions associated with attention during successful response inhibition or craving resistance (Volkow et al., 2010; O’Connor et al., 2012). These regions include the right superior temporal gyrus (rSTG), which has been associated with shifting attention in tasks that involve relative value coding (Hampton et al., 2008; Hare et al., 2010; Lim et al., 2013).

We have previously argued that the ability to direct attention away from an alluring stimulus may be an endogenous mechanism that assists inhibitive control over that stimulus when there is a reason to do so (O’Connor et al., 2012). If an individual can direct attention away from a cue, then the prepotency of an alluring stimulus is reduced and inhibition may be easier, requiring less involvement of rIFG than might otherwise be the case (O’Connor et al., 2012). During employment of cognitive control strategies designed to resist cue-induced craving, hypoactivation of visual processing areas was accompanied by either no significant change to rIFG activation (Brody et al., 2007) or rIFG hyperactivity (Volkow et al., 2010). The results may be further evidence that for improved understanding of PFC-related resistance to reward, attentional mechanisms should be taken into account.

In this functional magnetic resonance imaging (fMRI) study, we modified a Go/No-go task to investigate whether response inhibition can be enhanced by exogenously diverting attention away from immediately rewarding target stimuli. In addition to standard Go trials requiring a button press response, we also introduced Go-Money trials which, when responded to with sufficient speed, provided immediate feedback for a small monetary reward. A subset of Go-Money trials was visually modified to indicate that participants had to inhibit their standard rapid rewarding responses (No-go trials). A response to No-go trials was considered a failure of response inhibition. Crucially, No-go trials could be accompanied by either a high or low means of diverting attention. This manipulation allowed us to investigate whether diverting attention from an immediately rewarding stimulus improves response inhibition. Processing faces appears to be automatic and mandatory (Farah, 1996; Vuilleumier and Schwartz, 2001; Lavie et al., 2003) and were therefore chosen to exogenously divert attention away from No-go trials. To contrast these high-level attention diversion No-go trials, scrambled faces accompanied the remaining No-go trials. Because scrambled faces do not possess the same attention-capturing characteristics, these served as low-level attention diversion No-go trials. Finally, No-go trials were further manipulated to address another important question. Specifically, if diverting attention away from immediately rewarding stimuli is actually effective in improving response inhibition, is this enhancement only effective if there is a larger potential reward available in the future? To explore this possibility, we compared performance for No-go trials where successful response inhibition would contribute to a larger reward but where no immediate feedback is given, against performance on No-go trials where no such delayed reward contingencies were available. In this way, the paradigm was designed to include a model of real-life circumstances in which abstinence is required over an immediate and tangible reward, in order to obtain a larger, less tangible, future benefit. In parallel with a hypothesized behavioral improvement in response inhibition over immediately rewarding stimuli as a result of attention diversion, we hypothesized modulation of brain regions relevant to cognitive control (PFC), reward (striatum), and attention (STG).

## Materials and Methods

### Participants

Twenty-six volunteers participated in this study, recruited from the community through advertising. Five participants were excluded from data analyses due to either non-completion of scan (one), excessive head movement during structural scans (three), or identification of anomalous anatomical features (one). Twenty-one healthy volunteers (13 females and eight males; *M*_age_ = 24.7 years, *SD* = 4.9, range = 17.7–33.5) were included in the data analyses. All were right-handed, as determined by the Edinburgh Handedness Inventory (Oldfield, 1971), and reported no current or past history of neurological or psychiatric disorders or psychotropic medication use. All participants, and a parent or guardian for those aged less than 18 years, provided written informed consent and were screened for physical or medical conditions affecting eligibility for magnetic resonance imaging (MRI) scanning. The University of Melbourne Human Ethics Committee approved the study protocol. Participants were compensated $20/h for their participation, plus 5% of the amount earned during the Go/No-go task. The average performance bonus was $34.

### Go/No-go Task

#### Key Condition Manipulations

**FIGURE 1:**
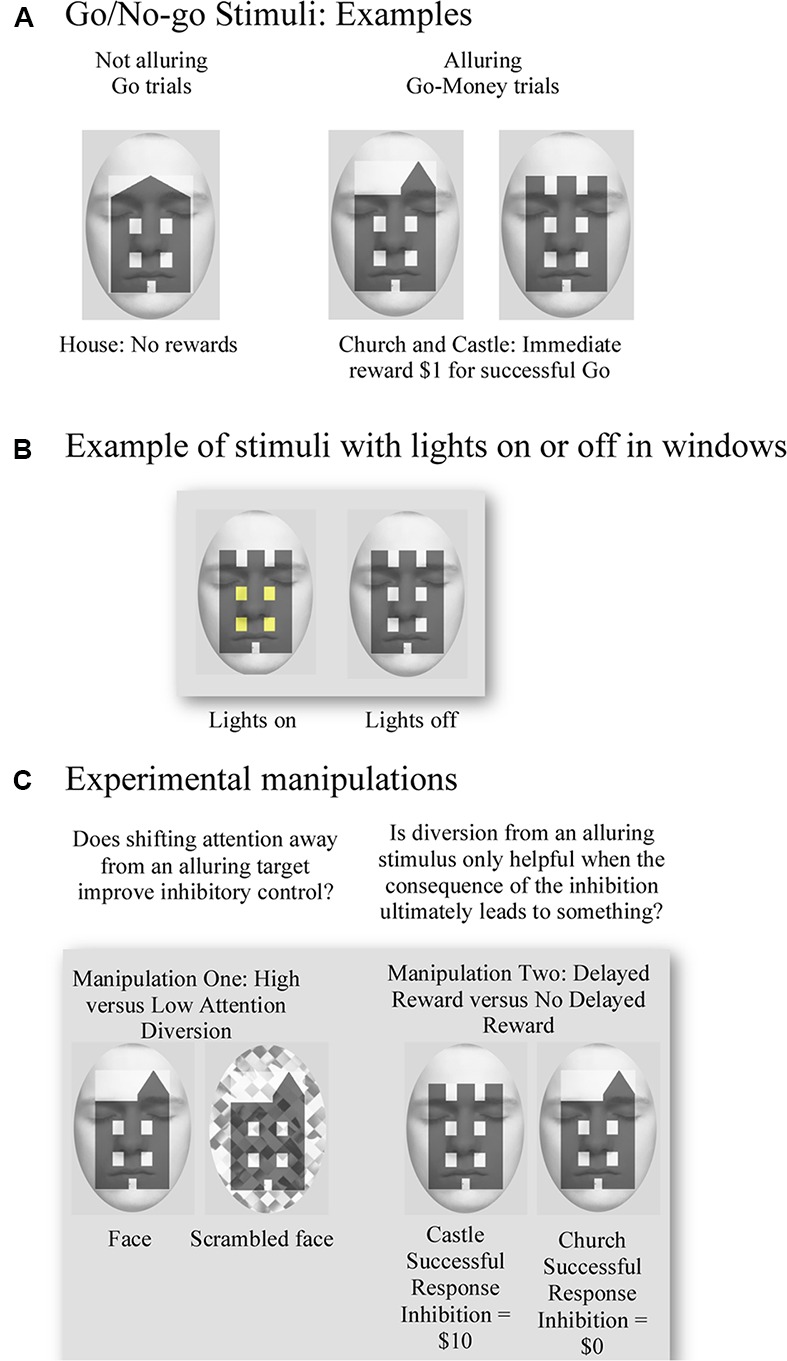
**Examples of the visual stimuli used for the Go/No-go task and experimental manipulations. (A)** Examples of target stimuli. No reward was associated with successful response to house (Go) trials, and an immediate one dollar reward was provided for successful response to church or castle (Go-Money) trials. **(B)** Example of use of lights for target stimuli. No-go trials were defined by whether a church or castle had lights on/off in their windows. **(C)** Examples illustrate manipulation of attention diversion by using a face and scrambled face in the background as high and low attention diversion, respectively. Successful response inhibition of a castle resulted in a ten-dollar reward, whereas successful response inhibition of a church yielded no reward.

The modified Go/No-go task consisted of two key condition manipulations (**Figure 1**). First, we examined the notion that diversion of attention away from an alluring stimulus might facilitate response inhibition by utilizing the assumed attention-grabbing attributes of faces. Second, we utilized a manipulation of delayed reward to test whether the proposed shift in attention away from immediately rewarding targets to aid response inhibition is exclusive to situations in which successful control of impulses yields a future benefit. The task manipulated future reward insofar as for half of the No-go events, successful response inhibition produced no delayed reward. In addition to these key manipulations, we also developed alluring reward-response associations for targets by providing small immediate monetary rewards and feedback for successful Go-Money and unsuccessful No-go trials.

For each trial, participants were presented with an image of a building. Two characteristics of the building stimulus determined whether participants should respond (Go trials), respond rapidly for a reward (Go-Money trials) or withhold their response (No-go trials). These characteristics were the building type (house, church, or castle) and whether lights were on or off in the windows of the building. Go trials, represented by houses, always required a response from participants. Neither reward nor performance feedback was provided for Go trials, so the target was not alluring. The second most frequent trials to appear, Go-Money trials, were represented by churches and castles. Upon presentation of Go-Money trials, participants were required to respond rapidly. A rapid response ensured immediate monetary reward feedback of $1 and meant that Go-Money trials were alluring. Finally, the least frequent type of trial, No-go trials, were also represented by churches and castles. This conflation with Go-Money trials was intentional as it meant that associations with immediate reward feedback were held constant and, therefore, No-go trials remained alluring. Indeed, failure to inhibit No-go trials still led to an immediate reward outcome of $1. The only characteristic that differentiated Go-Money trials from No-go trials was whether the windows of the building showed lights to be “on” or “off.” Therefore, depending on instructions at the beginning of the experimental block, participants could respond rapidly to presentation of a church with lights “on” in one trial and then withhold response to presentation of a church with lights “off” in another trial. This conflation and nuanced difference also ensured that participants had to attend fully to each stimulus in order to attain the appropriate behavior.

As previously outlined, experimental conditions varied on two factors: (a) high and low attention diversion; and (b) large delayed reward and no delayed reward for successful response inhibition. For (a) high and low attention diversion, stimuli were presented with a background face or scrambled face, respectively. Although we were only interested in the effect of attention diversion on No-go performance, face/scrambled face backgrounds were applied to all trials to ensure that participants could not identify No-go trials simply by the presence of a face or scrambled face. To minimize emotional engagement and possible related interference in amygdalae, a passive face with eyes closed was used. Faces were fitted to an oval frame and placed behind relevant stimuli. For (b) large delayed reward and no delayed reward for successful response inhibition, the concept of a “delayed” or future reward was simulated through the absence of any immediate feedback for successful response inhibition. In this way, the requirement to inhibit a response was not tangibly alluring, as it provided no concrete feedback about reward outcomes. Moreover, both delayed and no delayed reward conditions had the same potent immediate reward association, so that failure to withhold an impulse yielded the previously learned immediate and tangible reward. Instead, participants were simply instructed that for each successful response inhibition of a No-go trial represented by a castle, they would earn a $10 reward, whereas each successful response inhibition of a No-go trial represented by a church would earn no such reward. Therefore, the condition of delayed reward for successful inhibition was modeled to be closest to a real-life situation, including the contingency that failure to abstain yielded some immediate, small reward. That is, abstinence was required over an immediate and tangible reward to obtain a larger, but less tangible, future benefit.

#### Task Design

**FIGURE 2:**
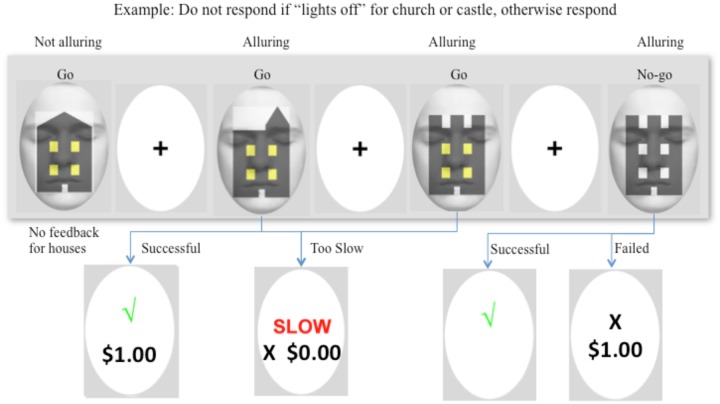
**Example of a typical sequence of the Go/No-go task.** In this example, participants were instructed to withhold their response when a “castle” and “church” had lights “off” in their windows. The first target was a house, which required a “Go” response at all times. No reward or performance feedback was provided for the house and so the target was not alluring. The following two figures were a church and castle with lights “on,” and both required a “Go” response. Each stimulus was presented for 750 ms, followed by a 1000 ms interstimulus interval, and then a 500 ms fixation cross. For Go-Money and No-go trials, the fixation cross was preceded by a feedback screen for 750 ms. A successful response provided immediate one dollar reward and feedback, and a response that was either too slow (>400 ms) or too fast (<100 ms) earned no reward although feedback was provided. The images were alluring because of the immediate one dollar reward. The fourth image was a castle with lights off, and therefore, response was to be withheld. Successful inhibition to the castle yielded a tick and a 10-dollar reward (or no reward for a church). No feedback was provided on monetary amount for delayed reward until the end of the session. Failed inhibition of castle or church resulted in a cross to signify the response was incorrect and immediate reward of one dollar.

Prior to entering the MRI scanner, participants were given detailed instructions and were fully practiced on the task and its contingencies until they had a good understanding of it. The Go/No-go task consisted of eight blocks of trials with 180 trials per block, and used a ratio of 6:2:1 Go:Go-Money:No-go responses (i.e., 120 Go, 40 Go-Money, and 20 No-go trials per block). In four of the eight blocks, Go-Money trials were defined as churches and castles with their lights “on,” and No-go trials were churches and castles with lights “off.” In the other four blocks, Go-Money trials were churches/castles with lights “off,” and No-go trials were churches/castles with lights “on.” An event-related design permitted presentation of different trials in arbitrary sequences, thus reducing potential for habituation or anticipation (Rosen et al., 1998), and facilitated averaging across specific events. Background attention diversion and target stimuli were counterbalanced across the present experiment such that there were four sequences in total. No-go trials were pseudo-randomly interspersed throughout the Go and Go-Money trials. The stimulus was presented for 750 ms, followed by a 1000 ms interstimulus interval, and then a 500 ms fixation cross. For Go-Money and No-go trials, the fixation cross was preceded by a feedback screen for 750 ms. In terms of feedback, a tick (√) or cross (X) was used to signify whether a response was correct or incorrect, respectively. Successful Go-Money trials, necessitated that response be within a time window of 100–400 ms. A response faster than 100 ms indicated high likelihood that the response preceded visual processing of the target (Macknik and Livingstone, 1998), and the 400 ms threshold was set to facilitate a spontaneous response. For Go-Money trials, participants received immediate feedback comprising a tick and one dollar reward for successful trials (√ $1.00) or that their response either took longer than 400 ms (Slow X $0.00), or was faster than 100 ms (Fast X $0.00). Successful withholding of a response to the castle yielded a tick and a 10-dollar reward although no feedback on amount earned for delayed reward was provided until the end of session. Failed inhibition of No-go trials resulted in immediate reward of one dollar (“X $1.00”). There was neither feedback nor reward for House trials. An example of a typical sequence is shown in **Figure 2**. This portion of the task was approximately 60 min in duration.

To mitigate possible “reversal learning” effects from the light on/off manipulation between blocks of Go/No-go trials, each block was preceded by a “Go task” which required participants to respond to all (Go-Money and Go) targets, within 100–400 ms. Each inter-block Go task was approximately 1 min in duration, and contained all stimuli in random order, with each target presented for 750 ms, followed by a feedback screen for 750 ms for Go-Money trials, and then a fixation cross for 500 ms. For Go-Money trials, participants received immediate feedback comprising a tick and one dollar reward for successful trials (√ $1.00) or that their response either took longer than 400 ms (Slow X $0.00), or was faster than 100 ms (Fast X $0.00). There was neither feedback nor reward for House trials.

#### Apparatus

Visual stimuli were presented using E-Prime software (version 2.0, Psychology Software Tools, Pittsburgh, PA, USA) on a laptop PC (Intel 2 Ghz, 256 mb Nvidia Video Card) that was interfaced with the magnetic resonance scanner during fMRI data acquisition. Stimuli were projected onto a screen located near the feet of the participant, who viewed the screen via a mirror attached to a 32 channel head coil. Behavioral responses were recorded using a scanner-compatible two-button box (Fiber-Optic response pad, Current Designs, Philadelphia, PA, USA). fMRI data were acquired at Swinburne University (Hawthorn, Australia) using a Siemens Tim Trio 3T MRI scanner (Erlangan, Germany).

### Functional Magnetic Resonance Imaging Data Acquisition

Functional magnetic resonance imaging data for the Go/No-go tasks were acquired using 180 (gradient) echo-planar imaging (EPI) sequences which provided T2^∗^ (time constant for loss of signal in sequence). Weighted BOLD activity measurements were obtained for each functional run with the following parameters: repetition time (TR) = 2 s; echo time (TE) = 36 ms; flip angle (FA) = 90°; 192 mm field of view; and 38 contiguous slices of 4 mm slice thickness. An oblique 30° orientation to the anterior–posterior commissure line was employed in data acquisition. The first two volumes of each run were discarded prior to data analysis to account for transients in the magnetic field of scanner. Eight functional runs were collected for each participant. At the completion of functional neuroimaging, high-resolution structural images were acquired using an MPRAGE T1-weighted sequence [TR = 1900 ms; TE = 2.3 ms, FA = 90, slice thickness = 0.90 mm; in-plane resolution = 1 mm × 1 mm]. During data processing, functional data were overlaid on the structural image for each participant, so that activations could be accurately localized with anatomy.

### Data Processing and Analysis

#### Go/No-go Data

Behavioral data was assessed using E-Prime 2.0 software (Psychology Software Tools, Pittsburgh, PA, USA), IBM^®^ SPSS^®^ Statistics Version 18 (Chicago, IL, USA), and Microsoft Excel (2007). To assess whether attention diversion improved response inhibition, and whether the outcome of the inhibition influenced performance, a two-way repeated measures analysis of variance (with pair-wise comparisons for significant effects) was conducted. The *F*-statistic was used to determine whether there was a significant difference between means. Confidence intervals were calculated using SPSS and provided additional information regarding the statistical power of each comparison. The dependent variable, mean response inhibition accuracy, was varied by two factors (a) level of attention diversion (High/Low), and (b) future reward (Delayed reward/No delayed reward). Additional secondary analyses were conducted to check for use of cognitive appraisal strategies during response inhibition (e.g., differences in response times between conditions and ensure a minimum level of responding).

#### Functional Magnetic Resonance Imaging Data

Functional magnetic resonance imaging data were processed using Analysis of Functional NeuroImages (AFNI) software (Cox, 1996). Following image reconstruction and concatenation of runs, functional data were time-shifted using Fourier interpolation to adjust for difference in slice acquisition times, aligned to corresponding anatomical data, and warped to standard Talairach space. Motion was corrected using three-dimensional volume registration with the third volume from the first run as a base. Volumes were blurred using a 4.1 mm full-width half max filter, each voxel was then scaled to a mean of 100 and values over 200 were clipped. To examine the influence of reward and attention diversion on inhibition performance, an event-related analysis was performed that estimated BOLD activity during correct No-go trials. Hemodynamic response functions were calculated using deconvolution of each successful No-go trial response. Activity related to errors, Go-Money trials, feedback screens and motion, were modeled as additional regressors to avoid contamination of baseline and event-related data. TR pairs were censored when the Euclidian Norm of the motion derivative exceeded 1.0. The baseline estimate was the mean activation recorded during the ongoing trial period (Go trials). Thus, the activation observed during successful No-go trial and Go-Money responses represented activation that differed from that required for the ongoing trial period (or Go) responses. The absence of collinearity between regressors within AFNI X-matrices was confirmed during deconvolution using xmat_tool.py. Event-related map voxels for each regressor of interest were extracted, resampled to anatomical data resolution (1 mm^3^), and masked using a group-averaged EPI mask dataset. Group activation maps for successful response inhibition were determined with one-sample *t*-tests against the null hypothesis of zero event-related activation changes (i.e., no change related to baseline). Significant voxels passed (a) a voxelwise statistical threshold (*t* = 4.84, *p* ≤ 0.0001), and (b) a continuity threshold – part of a larger 112 μl cluster of contiguous significant voxels. The combination of probability and cluster thresholding maximized the power of the statistical test and minimized the likelihood of false positives. Simulation using the 3D ClustSim function (run within the whole brain) in AFNI and an uncorrected voxelwise threshold *p* = 0.0001, indicated a minimum cluster size of 112 μl. Activation clusters derived from this whole brain analysis of response inhibition were used to construct activation maps. Whole brain analysis revealed regions of event-related activation during successful No-Go trials. The mean activation for clusters in this map was calculated for the purposes of a functionally derived region of interest (ROI) analysis. ROIs were functionally defined by the No-go > Go contrast. Activation estimates between conditions were compared using two-way repeated-measures ANOVA, *post hoc* pairwise comparisons tested the effects of our experimental manipulations, and corrected using a modified Bonferroni procedure for multiple comparisons (Keppel, 1991).

## Results

### Behavioral Data

**FIGURE 3:**
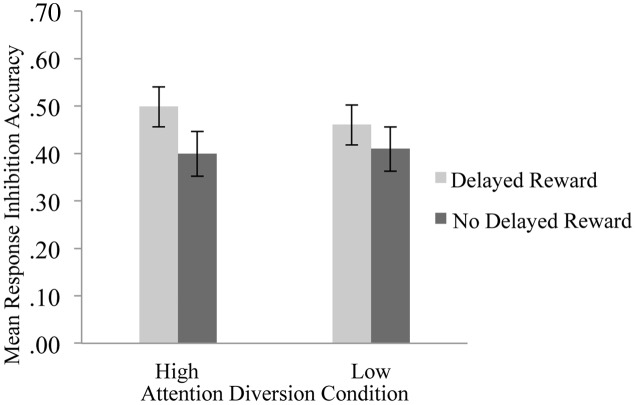
**Mean response inhibition accuracy by strength of attention diversion and delayed reward conditions.** Error bars represent standard error of the mean. *n* = 21. Attention diversion assisted response inhibition performance, but only when inhibition led to delayed gratification.

The effect of attention diversion on mean response inhibition accuracy in delayed reward and no delayed reward trials of the Go/No-go is shown in **Figure 3**. There was a significant interaction effect between reward and attention diversion condition on inhibition accuracy, *F*(1,20) = 6.14, *p* = 0.022, $\eta_{p}^{2}$ = 0.24. For delayed reward trials, mean inhibition accuracy was significantly higher in the high attention diversion condition, *M* = 050, *SD* = 0.19, 95% CI [0.41, 0.58], than in the low attention diversion condition, *M* = 0.46, *SD* = 0.20, 95% CI [0.37, 0.55], but not significantly different for no delayed reward trials, high attention diversion condition, *M* = 0.40, *SD* = 0.21, 95% CI [0.30, 0.50], and low attention diversion condition, *M* = 0.41, *SD* = 0.22, 95% CI [0.31, 0.51]. Main effects of delayed reward, *F*(1,20) = 7.44, *p* = 0.013, $\eta_{p}^{2}$ = 0.27, and strength of attention diversion, *F*(1,20) = 0.72, *p* = 0.406, $\eta_{p}^{2}$ = 0.04, were qualified by the significant interaction effect. In summary, attention diversion assisted response inhibition performance, but only when response inhibition led to future reward.

We also examined whether response inhibition improvements were in part due to trade-offs in Go and Go-Money trial response times (RTs). For alluring Go-Money and non-alluring Go trials (houses), overall mean RTs were faster for high attention diversion (face) and non-alluring stimuli (house) conditions (**Table 1**). There was no significant interaction effect between reward and attention diversion condition on RTs, *F*(1,20) = 2.22, *p* = 0.152, $\eta_{p}^{2}$ = 0.10, and no main effect of strength of attention diversion, *F*(1,20) = 0.96, *p* = 0.338, $\eta_{p}^{2}$ = 0.05. The main effect of reward condition was significant, *F*(1,20) = 10.15, *p* = 0.005, $\eta_{p}^{2}$ = 0.34. For low attention diversion trials, RTs were significantly faster for Go trials than for Go-Money trials, *mean difference* = 6.79 ms, *p* = 0.013, 95% CI [1.60, 11.99], and for high attention diversion trials, RTs were also significantly faster for Go trials than for Go-Money trials, *mean difference* = 11.55 ms, *p* = 0.008, 95% CI [3.35, 19.76]. In summary, mean RTs were faster for Go trials than Go-Money trials. In addition, this difference did not vary as a function of attention diversion strength suggesting accuracy data was representative of response inhibition performance.

**Table 1:** Mean response times in milliseconds by stimuli and attention diversion.

Condition	Mean	*SD*	95% CI
Go-Money (Churches and Castles)/Face	381.7	55.1	[356.7, 406.8]
Go-Money (Churches and Castles)/Scrambled Face	388.5	54.5	[363.7, 413.3]
Go (Houses)/Face	373.7	41.2	[354.9, 392.5]
Go (Houses)/Scrambled Face	385.2	42.3	[366.0, 404.5]

*n = 21. SD, standard deviation; CI, confidence interval*.

### Functional Magnetic Resonance Imaging BOLD Activity

Whole brain analysis revealed 20 regions of event-related activation during successful No-go trials (as shown in **Table 2**). Event-related ROI analysis was, however, restricted to *a priori* neural areas (STG, rIFG, and striatum) to avoid reverse inferences (as recommended by Kriegeskorte et al., 2009). Previous literature, including our recent work, has shown these nodes to be implicated in shifts of attention during response inhibition (STG; O’Connor et al., 2012; Lim et al., 2013), goal-directed stopping (rIFG; Aron, 2011), and dorsal striatum in reward-related processes for goal-directed behavior (O’Connor et al., 2012). Therefore, subsequent ROI analyses focussed on the activity of these selected clusters, which were identified as functionally relevant. Also, minimizing the number of ROIs reduced the probability of a type I error (Poldrack, 2007).

**Table 2:** Regions of event-related activation during successful No-go trials.

			Center of Mass^c^		
Structure	HS^a^	Volume (μl)^b^	*x*	*y*	*z*
Insula	R^d^	1888	32	17	12
Insula	L^e^	2052	-29	16	14
Superior Frontal Gyrus	R	1652	13	48	44
Medial Frontal Gyrus^∗^	R	999	2	4	54
Medial Frontal Gyrus	L	906	-2	46	46
Postcentral Gyrus^∗^	L	864	-49	-19	23
Middle Frontal Gyrus	R	133	36	5	58
Middle Frontal Gyrus	L	600	-36	4	59
Inferior Frontal Gyrus^∗∗^	R	352	41	-5	38
Inferior Frontal Gyrus^∗^	L	550	-41	0	39
Caudate^∗^	L	524	-14	5	5
Fusiform Gyrus	R	615	25	-59	-8
Fusiform Gyrus	L	279	-25	-70	-6
Inferior Parietal Lobule	R	284	44	-70	44
Posterior Cingulate	R	261	1	-56	28
Culmen	R	188	12	-69	-2
Cingulate Gyrus^∗^	L	182	0	-44	42
Superior Temporal Gyrus^∗^	R	149	59	-37	10
Superior Temporal Gyrus^∗∗^	L	160	-61	-25	2
Parahippocampal Gyrus	R	113	18	-42	8

*n = 21. ^a^HS, hemisphere; ^b^μL, microliters. ^c^Montreal Neurological Institute Brain Atlas Anatomical Coordinates. ^d^R, right; ^e^L, left. ^∗^p < 0.05; ^∗∗^p < 0.01. Sidak corrected. The following structures had significant main effects for distractor conditions: left inferior frontal gyrus, caudate, and right and left superior temporal gyri. The following structures had significant main effects for delayed-reward conditions: right inferior frontal gyrus, right medial frontal gyrus, left postcentral gyrus, and left cingulate gyrus*.

**FIGURE 4:**
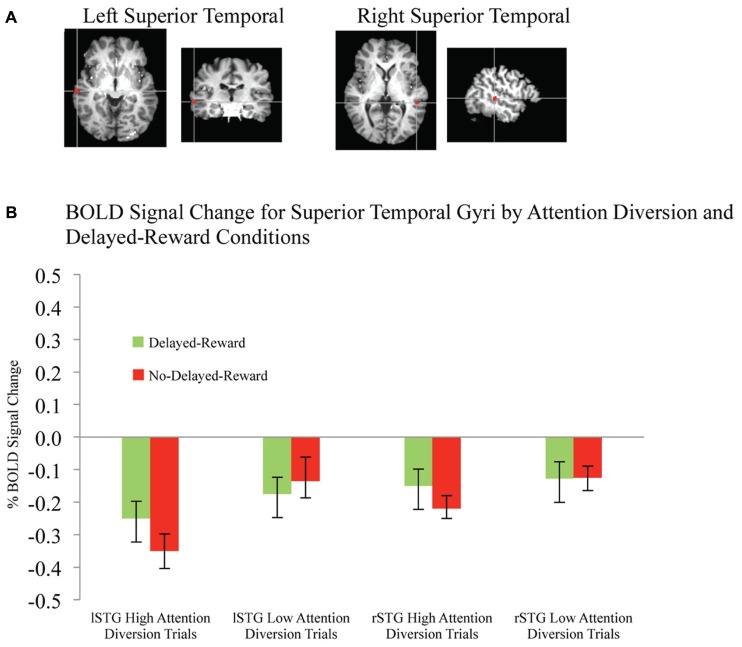
**BOLD signal change in superior temporal gyri during successful response inhibition. (A)** Activation maps for left and right superior temporal gyri showing significant activity for successful response inhibition, overlaid on coronal brain sections (Talairach template; using AFNI software), *p* ≤ 0.0001. **(B)** Change in BOLD activity plotted by attention diversion and delayed reward conditions. Error bars represent the standard error of the mean. *n* = 21.

**FIGURE 5:**
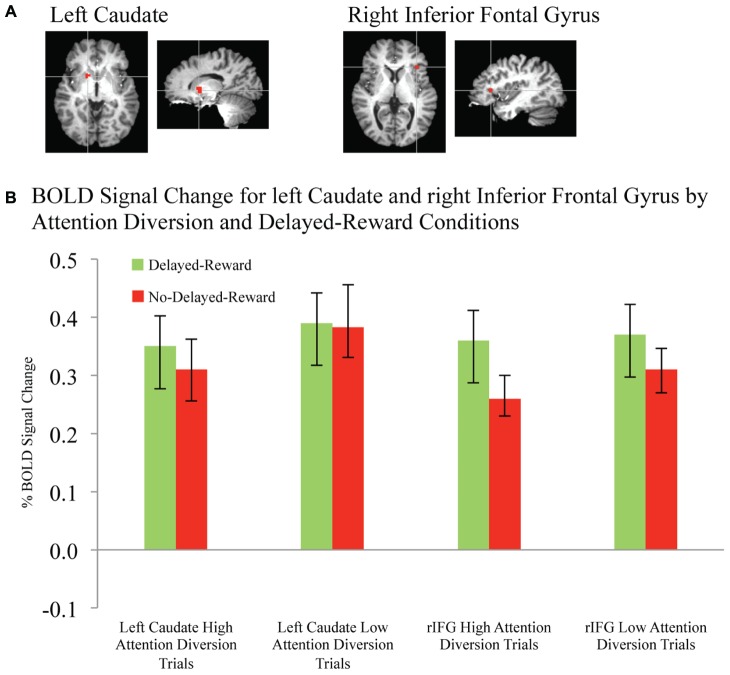
**BOLD signal change in left caudate and right inferior frontal gyrus during successful response inhibition. (A)** Activation maps for left caudate and right inferior frontal gyrus showing significant activity for successful response inhibition, overlaid on coronal brain sections (Talairach template; using AFNI software), *p* ≤ 0.0001. **(B)** Change in BOLD activity plotted by attention diversion and delayed reward conditions. Error bars represent the standard error of the mean. *n* = 21.

Activation maps and percentage BOLD signal change for the STG, left caudate, and rIFG are shown in **Figures 4** and **5**, and statistical analysis is summarized in **Table 3**. There was no significant interaction effect between reward and attention diversion condition on any of rSTG, *p* = 0.135, left STG (lSTG), *p* = 0.121, left caudate, *p* = 0.451, or rIFG activation *p* = 0.428. Although the interaction effects were non-significant, the behavioral data revealed a context specific effect of our attentional manipulation such that high attention diversion only facilitated inhibitory control when paired with the possibility of delayed reward. In order to assess the neural bases of this effect, pairwise comparisons were conducted. The main effect of delayed reward condition was not significant for rSTG activation, *p* = 0.134, lSTG, *p* = 0.121, nor left caudate, *p* = 0.383, but was significant for rIFG activation, *p* = 0.001. The main effect of attention diversion strength on rIFG activation was not significant, *p* = 0.377, but was significant for rSTG activation, *p* = 0.029, lSTG, *p* < 0.001, and left caudate, *p* = 0.030. For the rSTG, for delayed reward trials activation was not significantly different between the high and low attention diversion conditions, *p* = 0.453, but activation was significantly lower in the high attention diversion condition than the low attention diversion condition for no delayed reward trials, *p* = 0.022. For the lSTG, for no-delayed-reward trials, activation was significantly lower in the high than low attention diversion condition, *p* = 0.004, and for delayed reward trials, activation was also significantly lower in the high attention diversion condition than the low attention diversion condition, *p* = 0.012. For the left caudate, activation was not significantly different between attention diversion conditions for delayed reward trials, *p* = 0.229. For no delayed reward trials, left caudate activation was significantly lower in the high attention diversion condition than the low attention diversion condition, *p* = 0.029.

**Table 3:** Region of interest analysis statistical summary.

Region	Test stastic *F*(1,20)	*p*-value^a^	Partial eta squared	Mean difference [95% CI]
**Left Superior Temporal Gyrus, coordinates^b^*xyz*, -61 -25 2, volume 160 μl**				
Interaction effect	0.58	0.121	0.12	
Main effect for delayed reward	2.63	0.121	0.12	
Main effect for strength of attention diversion (AD)	27.14	<0.001	0.58	
No delayed reward condition (High less Low AD strength)		0.004		-0.18 [-0.29, -0.06]
Delayed reward condition (High less Low AD strength)		0.012		-0.12 [-0.21, -0.03]
**Right Superior Temporal Gyrus, coordinates^b^*x y z*, 59-37 10, volume 149 μl**				
Interaction effect	2.42	0.135	0.11	
Main effect for delayed reward	2.44	0.134	0.11	
Main effect for AD strength	5.55	0.029	0.22	
No delayed reward condition (High less Low AD strength)		0.022		-0.95 [-0.17, -0.02]
Delayed reward condition (High less Low AD strength)		0.453		-0.02 [-0.08, 0.04]
**Left Caudate, coordinates^b^*x y z*,-14 5 5, volume 524 μl**				
Interaction effect	0.59	0.451	0.03	
Main effect for delayed reward	0.80	0.383	0.04	
Main effect for AD strength	5.43	0.030	0.21	
No delayed reward condition (High less Low AD strength)		0.029		-0.07 [-0.14, -0.01]
Delayed reward condition (High less Low AD strength)		0.229		-0.04 [-0.11, 0.03]
**Right Inferior Frontal Gyrus, coordinates^b^*x y z*, 59-37 10, volume 352 μl**				
Interaction effect	0.66	0.428	0.03	
Main effect for delayed reward	0.82	0.377	0.04	
Main effect for AD strength	14.21	0.001	0.42	
No delayed reward condition (High less Low AD strength)		0.080		0.06 [-0.01, 0.13]
Delayed reward condition (High less Low AD strength)		0.003		0.09 [0.04, 0.15]

*n = 21. CI, confidence interval. ^a^Sidak corrected; ^b^Peak Montreal Neurological Institute Brain Atlas Anatomical Coordinates*.

In summary, deactivation of the rSTG was significantly greater for high than low attention diversion condition, but only for no delayed reward trials, and deactivation of the lSTG was significantly greater for the high than low attention diversion condition, for all successful No-go trials. Activation of the left caudate was significantly lower for high than low attention diversion condition, but only for no delayed reward trials, and activation of the rIFG was significantly greater for delayed reward trials than no delayed reward trials, but only for the high attention diversion condition.

## Discussion

In this study, we used exogenous diversions of attention to vary the degree to which participants directed their attention away from alluring stimuli. We found that attention diversion improved response inhibition performance. This finding adds to previous research by providing evidence that response inhibition can be improved by exogenously modulating attention. We also manipulated future benefit in that successful withholding either led to a larger, delayed reward or no delayed reward. Interestingly, we found that attention diversion was only helpful to response inhibition performance, when successful control of impulses ultimately led to a larger, delayed reward. Lack of perceived benefit may, therefore, compromise the application of implicit self-control behaviors.

Regions previously implicated in response inhibition and attention showed sensitivity to context-specific manipulations of attention. Specifically, we observed increased recruitment of the posterior rIFG and enhanced response inhibition performance for delayed reward trials compared to no delayed reward trials. Engagement of posterior rIFG has been associated with selective, goal-directed stopping (Chikazoe et al., 2009; Verbruggen et al., 2010; Aron, 2011). rIFG activation did not vary across the attention diversion conditions. However, STG were more de-activated for high attention diversion trials than low. Increased deactivation of STG is interpreted to be indicative of a degree of disengagement from salient (immediately rewarding) stimuli, facilitating successful response inhibition (O’Connor et al., 2012).

Shifts of attention away from target stimuli might also be reflected in reduced activity in reward-related neural processes. Our finding of increased deactivation of the left caudate for higher attention diversion trials was consistent with this concept. Dorsal striatal activity has been associated with elevated target saliency (Pizzagalli et al., 2009; Onoda et al., 2011), and computation of relative value between outcomes or in comparison to expected reward (O’Doherty et al., 2004; Balleine and O’Doherty, 2010; Wunderlich et al., 2012). The inference is that the encoding of value potentially informs prefrontal executive processes, consistent with a system that computes value and best action-outcome (Frydman et al., 2011). However, our left caudate results were only significant for the no delayed reward condition and not the delayed reward condition. Other regions, such as the ventral striatum and orbitofrontal cortex, have been consistently linked to reward-related processes (Fitzgerald et al., 2009; Plassmann et al., 2010). Comparison of BOLD activity between reward and attention diversion conditions did not detect any significant change in these regions during successful response inhibition. However, the context for reward in our study is different, which may explain why the conditions do not vary.

Our findings may have relevance in a number of health issues where impaired self-control is thought to be factor. Some clinical populations are known to exhibit compromised response inhibition and high reward sensitivity (e.g., addiction, Goldstein and Volkow, 2002; Dackis and O’Brien, 2005). Deficits in using shifts of attention to diffuse target saliency may be critical for the initiation and continuation of self-regulatory lapses (e.g., rumination in residual depression and relapse associated with exposure to conditioned drug-cues in addiction). It would be instructive to understand whether differences in these self-regulatory processes between individuals vary over time (Kane and Engle, 2002; Unsworth and Engle, 2007; Friedman et al., 2008; Casey et al., 2011), are reflected in neural circuitry or functioning of the dopaminergic network (Buckholtz et al., 2010), and amenable to training (Klingberg, 2010). Our findings indicate that perceived lack of future benefit may undermine the application of such implicit self-control behaviors. In public health policy, measures to diffuse target saliency and emphasize future benefit may aid resistance to immediate reward. For instance, plain paper packaging of cigarettes may help reduce cigarette saliency, and extolling the future health benefits of stopping smoking may provide a more tangible delayed reward for smokers. Also, a perception that successful resistance to an immediate reward brings uncertain future benefit may somewhat deplete response inhibition capacity, resulting in more impulsive behavior (Milkman, 2012).

The experimental design was a practical way to mimic real-world situations as the MRI scanner environment is highly constrained. However, a caveat is that our laboratory findings may not translate to everyday circumstances. Delay to reward was constrained by the duration of the laboratory session and might not be analogous to real-life delayed reward. The facilitating effect of faces on response inhibition may not generalize to other situations. While it is difficult to determine the authenticity of participants’ efforts to adhere to inhibition instructions or their willingness to engage properly in the exercise, analysis of performance markers (e.g., response times, accuracy, non-response to Go trials) indicated a high level of engagement and diligence. These results indicate that improved response inhibition accuracy on high attention diversion/delayed reward No-go trials cannot be attributed to slower response speed. In addition, people responded more quickly to Go-trials (houses) than Go-Money trials (churches and castles), which was expected given that identification of a single feature (building type for house) was sufficient to respond to the house, compared to two features for churches and castles (building type and lights). Also, high levels of non-response to Go-Money trials would have implied a strategy to maximize No-go gain, and similarly, relatively low accuracy (less than 10%) for No-go trials may have indicated difficulty with the task or a strategy of maximizing immediate rewards. There was, however, no evidence of strategies to maximize immediate or delayed reward. Moreover, block order was counterbalanced throughout the experiment to mitigate possible learning effects, and we used inter-block “Go task” runs to mitigate effects of reversal learning on subsequent blocks and reinforce immediate reward association irrespective of previous contingencies.

Future research could extend the present study by utilizing gradational changes to attention diversion (face) stimuli in order to expand understanding of the functional relationship between attention diversion, reward, and response inhibition. The methodology has been used successful in recent research (e.g., Preuschoff et al., 2008), but the high number of events necessary for sufficient power precluded integration of the methodology within the current study. In addition, a modified task comprising of explicitly stated temporal delays with real waiting periods before rewarding successful inhibition performance would lend greater credibility to the suggestion that this type of task represents a fusion of response inhibition (impulsive action) and delay discounting (impulsive choice).

This study adds to previous literature of the importance of an attentional mechanism for successful response inhibition of alluring stimuli. In the present experimental paradigm, inhibition appears to be a set of distinct neural processes related to stopping and the ability to implicitly control attention, which might be a target for intervention (training). This study also highlighted the importance of perceived future benefit for these implicit self-regulatory behaviors.

## Author Contributions

DO and FS designed the study, and collected, analyzed, and interpreted the data. FS drafted the manuscript, and DO and RH revised the manuscript. RH, CO, and KM contributed to study design. All authors discussed the results and commented on the manuscript.

## Conflict of Interest Statement

The authors declare that the research was conducted in the absence of any commercial or financial relationships that could be construed as a potential conflict of interest.
